# The effects of hindlimb unloading versus dietary cholesterol and resistance training on rat skeletal muscle responses

**DOI:** 10.1186/s12944-018-0944-9

**Published:** 2019-01-05

**Authors:** Teak V. Lee, Chang Woock Lee, Vincent C. W. Chen, Steve Bui, James D. Fluckey, Steven E. Riechman

**Affiliations:** 1 Life Sciences Department, Pierce College, 6201 Winnetka Avenue, Woodland Hills, CA 91371 USA; 2 School of Education, Health Professions, and Human Development, University of Houston-Victoria, Victoria, TX USA; 3 Exercise Science, Wellness and Sports Department, Georgian Court University, Lakewood, NJ USA; 4 Department of Health and Human Performance, Dixie State University, St. George, UT USA; 5 Department of Health and Kinesiology, Texas A & M University, College Station, TX USA; 6 Department of Nutrition, Texas A & M University, College Station, TX USA

**Keywords:** Hindlimb unloading, Dietary cholesterol, Resistance training, And proteins essential to cholesterol metabolism

## Abstract

**Background:**

The loss of muscle mass and concomitantly strength, poses a serious risk to the elderly and to astronauts. Dietary cholesterol (CL), in conjunction with resistance training (RT), has been strongly associated with improvements in lean mass. The purpose of this study was to examine the effects of two opposing environments on rat skeletal muscle: (1) hindlimb unloading and (2) CL and RT.

**Methods:**

In protocol 1, 13 male Sprague-Dawley rats were unloaded for 28 days (HU; *n* = 6) or served as cage controls (CC; *n* = 7). In protocol 2, 42 rats were assigned to 1 of 6 groups: CC (n = 7), CC + CL (*n* = 4), RT controls (RTC; n = 7), RTC + CL (*n* = 8), RT (n = 8) and RT + CL (n = 8). RT/RTC consisted of squat-like exercise. RT had weights added progressively from 80 to 410 g over 5 weeks. CL was supplemented in the chow with either 180 ppm (controls) or 1800 ppm (CL). Lower limb muscles were harvested at the end of both protocols and analyzed by Western Blotting for sterol regulatory element-binding protein-2 (SREBP-2) and low-density lipoprotein-receptor (LDL-R) and protein synthesis.

**Results:**

Gastrocnemius and plantaris masses and their body mass ratios were significantly lower in the HU rats than control rats. The RT rats gained significantly less body and lean mass than the RTC groups, but the plantar flexor muscles did not show any significant differences among groups. Moreover, RT groups had significantly higher plantaris mixed muscle fractional synthesis rate (FSR) than the RTC and CC animals, with the CL groups showing greater FSR than control rats. No significant differences among groups in SREBP-2 or LDL-R were observed in either protocol.

**Conclusions:**

These studies provide evidence for a relationship between skeletal muscle and cholesterol metabolism, but the exact nature of that association remains unclear.

## Background

The loss of muscle mass (sarcopenia) and concomitantly strength, poses a serious risk to the elderly as well as astronauts. Every decade starting around the age of 40, adults lose about 8% of their muscle mass, which may lead to greater body fat and higher risk for chronic diseases such as cardiovascular disease and diabetes [1, 2]. Following a short duration spaceflight of only 17 days, gastrocnemius and soleus muscle volume of astronauts decreased by ~ 10% each, which may negatively affect the successful completion of a mission [3]. Thus, there is a need to maintain and preferably increase muscle mass in these populations to minimize this risk.

Resistance training (RT) is an effective intervention that has shown success in increasing or maintaining muscle. Following an acute session of resistance exercise (RE), skeletal muscle protein synthesis rises above resting levels [4]. The addition of a supplement (amino acids and carbohydrates) after a single bout of RE augments this increase in protein synthesis [5]. Through repeated bouts of RE (resistance training) with and without supplementation, the elevated rates of protein synthesis may lead to muscle accretion, even in the elderly. Ten weeks of RT increased muscle mass and strength in the elderly while postmenopausal women increased lean mass and muscular strength following 24 weeks of RT with post-exercise dietary supplementation (protein and carbohydrate) [6, 7].

In addition to dietary supplementation with protein and carbohydrate, dietary cholesterol, in conjunction with RT, has been strongly associated with improvements in lean mass. We observed that older adults who performed 12 weeks of RT had significantly higher gains in lean mass with greater intakes of dietary cholesterol [8]. Moreover, greater low-density lipoprotein (LDL) cholesterol was also associated with larger gains in lean mass independent of dietary cholesterol [8].

Cholesterol is a critical constituent of all plasma membranes, including skeletal muscle sarcolemma, and provides membrane fluidity [9]. Additionally, cholesterol is a component of lipid rafts, which plays a role in cellular signaling involved with muscle growth [10]. One of the key proteins involved in cholesterol metabolism is sterol regulatory element-binding protein-2 (SREBP-2). SREBP-2 is a transcription factor that increases the expression of cholesterol biosynthetic enzymes and cholesterol uptake proteins (i.e. LDL receptor) (LDL-R) when intracellular sterol levels are low [11].

The purpose of this study was to examine the effects of two opposing environments on rat skeletal muscle: muscle-wasting, through an established animal paradigm (hindlimb unloading) (HU) mimicking spaceflight and disuse, and muscle-promoting with dietary cholesterol and RT [12–18]. We hypothesized that SREBP-2 and LDL-R protein expression in rat skeletal muscle would be reduced in the muscle-wasting condition while these proteins would be elevated in the muscle-promoting environment. Furthermore, we hypothesized that dietary cholesterol and RT would enhance skeletal muscle mass and rates of protein synthesis when compared to the cage control animals and rats performing RT only.

## Methods

### Protocol 1

#### Animals

Thirteen 6-month-old male Sprague Dawley rats were obtained from Harlan Laboratories (Houston, TX). Following 14 days of acclimation, the rats were allocated to individual cages in a climate and light regulated (12 h light/12 h dark cycle) room. Rats were given free access to a standard rodent diet (Harlan Teklad 8604) and water. Rats were then assigned to one of two groups based on body mass: cage control (CC, *n* = 7) or hindlimb unloading (HU, *n* = 6). All experimental procedures were approved by Texas A&M University’s Institutional Animal Care and Use Committee.

#### Hindlimb unloading

Rats in the HU group underwent 28 days of tail suspension [19]. Following anesthesia with isoflurane, the rat’s tail was cleaned with a brush and soap, rinsed, and dried with a towel. A gauze pad containing acetone was applied to the tail and a hair dryer was used to dry the tail. The tail was then covered with a quick drying adhesive (Cramer Q.D.A. tape adherent, Gardner, KS), which was allowed to dry before an additional adhesive was applied to the sides of the tail (Amazing Goop, Eclectic Products, Pineville, LA). A harness consisting of cloth tape, a bobby pin, a paper clip, and staples was placed over the second adhesive on the tail. After allowing approximately 30 min. for the second adhesive to dry, the animal was placed in an HU cage, and the harness was fixed to a pulley and cable apparatus that was situated across the top center region of the cage. This attachment was adjusted to create an approximately 30° head-down tilt in order to preclude the animal’s hindlimbs from touching the walls and floor of the cage, but to permit it to move freely with its forelimbs everywhere else.

#### Tissue harvest

After 28 days of HU, rats were anesthetized with an intraperitoneal injection of ketamine (50 mg/kg) and medetomidine (0.5 mg/kg), and the gastrocnemius and plantaris muscles were excised, weighed and frozen immediately in liquid nitrogen for subsequent analysis. Adrenal glands were also removed and wet weights were measured to determine the stress responses to HU.

#### Western blotting

Following pulverization in liquid nitrogen, approximately 40 mg of gastrocnemius and plantaris muscle was weighed and placed in individual vials with 270 μl of a lysis buffer [25 mM HEPES, 5 mM ß-glycerophosphate, 200 μM ATP, 25 mM Benzamidine, 2 mM PMSF dissolved in DMSO, 4 mM EDTA, 10 mM MgCl_2_, and 0.5% protease inhibitor (Sigma-Aldrich, St. Louis, MO)] and 30 μl of 10% Triton X-100. The samples were then homogenized, placed on ice for 1–1.5 h and centrifuged at 14,000 rpm at 4 °C for 30 min. The supernate, which contained the cytosolic and membrane-bound proteins, was saved for the bicinchoninic acid protein assay and electrophoresis [20].

Eighty micrograms of protein was obtained from each sample and mixed with Laemmli sample buffer. The samples were heated for 10 min and separated by SDS-PAGE for 101 min at 30 mA. The proteins from the gels were transferred to nitrocellulose membranes through a 3-step wet transfer protocol (1.3 mA/cm^2^ for 1 h, 3.8 mA/cm^2^ for 14 h, 7.5 mA/cm^2^ for 1 h). The membranes were blocked with non-fat milk in TBS (blocking buffer) for 1 h at room temperature, washed with TBS for 5 min for 3 replicates and then incubated with either SREBP-2 (Santa Cruz Biotechnology, Dallas, TX) or LDL-R (Abcam, Cambridge, MA) primary antibodies overnight at 4 °C. The membranes were subjected to a set of 3 TBS washes and then incubated with secondary antibody for 1 h at room temperature. After another series of 3 TBS washes, enhanced chemiluminescence (SuperSignal West Pico Chemiluminescent Substrate, Thermo Scientific, Rockford, IL) was used to detect (FluorChem Alpha Innotech) the protein bands of interest. The integrated density values (IDV) of the bands were normalized to the standard for the respective membranes and to the Ponceau stain as a loading control [21]. The values were reported in arbitrary units with the cage control values set at 1.

### Protocol 2

#### Animals and operant conditioning

Forty-two male Sprague-Dawley rats (age = 5–6 months) were purchased from Harlan Laboratories and resided in individual cages within a temperature and light regulated room at an animal housing facility. Following 7 days of acclimation, rats were randomized by body mass to one of the following groups: cage control (CC, *n* = 7), cage control + cholesterol (CC + CL, *n* = 4), resistance training control (RTC, n = 7), resistance training control + cholesterol (RTC + CL, *n* = 8), resistance training (RT, *n* = 8) or resistance training + cholesterol (RT + CL, n = 8). The Institutional Animal Care and Use Committee at Texas A&M University approved all experimental procedures.

Operant conditioning was used to train the RT and RTC groups to pull down on an illuminated bar situated high above the rat to turn it off. To achieve this movement, the rat needed to rise up on its hindlimbs to depress the switch. A foot shock of short duration (1 mA, 60 Hz) was utilized as negative reinforcement to promote this learning process. These animals performed this exercise for 4 sessions of 50 repetitions without any added resistance. Following these 4 sessions, an additional 2 bouts were completed with the rats performing the same protocol while wearing a non-weighted, leather and Velcro vest. Throughout the study, rats in the cage control groups did not perform any operant conditioning or RT.

#### Resistance training

During the study, the RT groups engaged in 5 weeks of progressive resistance training. Each week, 3 resistance exercise sessions were completed, which were separated by at least 48 h. For the 1st session, 80 g was added to the vest, and rats needed to perform 50 repetitions. Subsequently, weight was increased but repetitions were concomitantly reduced, resulting in total volume gradually decreasing by 5% per week such that the final session consisted of 16 repetitions with 410 g. The RTC groups performed similar training and experienced the same number of shocks as the RT groups; however, the RTC groups exercised without resistance.

#### Food

All rats were given free access to a standard rat chow diet (Purina Test Diet 5001). This diet consisted of 54% carbohydrates, 24% protein, 12% fat, 7% ash, 5% fiber and vitamins. However, at the start of the study, the CL groups were fed a similar chow that differed only in cholesterol levels (1800 ppm for CL vs. 180 ppm for standard chow).

#### Dual energy X-ray absorptiometry (DEXA)

At baseline and at the end of the study, DEXA (GE Lunar Prodigy) scans of the animals using a special program designed for rats were taken after administering anesthesia via Ketamine (40–55 mg/kg) and Medetomidine (0.4 mg/kg) in order to determine alterations in body composition.

#### Deuterium oxide for protein synthesis

Twenty hours before the final resistance exercise session, 1.5 ml of deuterium oxide was injected intraperitoneally into each rat. Animals were also given free access to drinking water containing 4% deuterium oxide to maintain enrichment levels [22].

#### Tissue harvest

After the rats were euthanized with Ketamine and Medetomidine, blood samples were obtained from the carotid artery, and then skeletal muscle (soleus, gastrocnemius, plantaris and quadriceps femoris) was harvested, weighed, and immediately placed in liquid nitrogen. Adrenal glands were also removed and weighed.

#### Fractional synthesis rates (FSR)

Plasma samples and standards were prepared and analyzed as described previously [23]. Briefly, 20 μl of plasma/standard, 4 μl of 5% acetone in acetonitrile (vol/vol), and 2 μl of 10 N NaOH were combined and permitted to settle for 24 h. Each sample/standard was thoroughly mixed with 600 μl of chloroform and 0.5 g of Na_2_SO_4_. The sample/standard was analyzed on a GC-MS (Agilent 5973 N-MSD furnished with an Agilent 6890 GC System and DB17-MS capillary column).

Approximately 30 mg of muscle (soleus and plantaris) from each sample was homogenized with 0.3 ml of 10% TCA and centrifuged for 15 min at 3800 rpm at 4 °C. The resultant pellet was then washed with 10% TCA, centrifuged, and disposed of the supernate for an additional 3 times. The pellet was then mixed with 6 N HCl and incubated for 18 h at 100 °C. The hydrolysate was then freeze dried for 24 h before 100 μl of a 3:2:1 ratio of methyl-8, methanol and acetonitrile were added to each sample and analyzed on the GC-MS.

The following equation was used to determine the mixed muscle protein FSR:

$$ {\mathrm{E}}_{\mathrm{A}}\cdotp {\left[{\mathrm{E}}_{\mathrm{BW}}\times 3.7\times \mathrm{t}\right]}^{\hbox{-} 1}. $$

E_A_ signifies the quantity of ^2^H-labeled alanine in protein (%), E_BW_ indicates the amount of ^2^H_2_O found in body water (%), and t represents time in h [23].

#### Western blotting

Quadriceps femoris and red gastrocnemius muscle samples were analyzed for SREBP-2 and LDL-R using the same protocol that was previously mentioned.

#### Statistical analysis

For protocol 1, all the data were analyzed using Statistical Analysis System (SAS) Enterprise Guide (v.7.1). An independent samples t-test was used to analyze differences between HU and CC groups. For gastrocnemius mature SREBP-2, adrenal mass and adrenal mass to body mass ratio, since these variables did not pass the Shapiro-Wilk Test of Normality, a Wilcoxon rank-sum test was used instead. For protocol 2, IBM (SPSS) Statistics 23 was used to analyze all the data. Two-way analysis of variance (ANOVA) was performed for activity and cholesterol groups. For both protocols, all the data were expressed as mean ± standard error (SE) and a *p*-value of < 0.05 was considered significant. Post hoc analysis with the Tukey-Kramer test was used to determine significant differences between groups in protocol 2.

## Results

### Protocol 1

#### Effect of 28 days of hindlimb unloading on body, muscle and adrenal mass

Following 28 days of unloading, there was no significant difference in body mass (*p* = 0.09) (Table 1), but there was a significant difference in the change in body mass between HU and CC groups (− 23.8 ± 2.8 g and 12.4 ± 3.7 g, respectively, *p* < 0.05). Gastrocnemius and plantaris masses and their body mass ratios were also significantly lower (p < 0.05) in the HU rats than control rats. Adrenal mass to body mass ratio (p < 0.05), but not adrenal mass (*p* = 0.08), was significantly higher in the HU rats than the CC rats.

**Table 1:** Effect of 28 days of hindlimb unloading on body, skeletal muscle and adrenal mass

	Cage Control (*n = 7*)	Hindlimb Unloading (*n = 6*)
Baseline body mass (g)	481.6 ± 15.0	478.8 ± 12.8
Post-study body mass (g)	494.0 ± 16.2	455.0 ± 12.1
Gastrocnemius (mg)	2553.8 ± 77.8	1907.1 ± 53.5^*^
Gastrocnemius to body mass (mg/g)	5.18 ± 0.09	4.20 ± 0.13^*^
Plantaris (mg)	535.7 ± 19.9	420.7 ± 15.7^*^
Plantaris to body mass (mg/g)	1.08 ± 0.02	0.93 ± 0.04^*^
Adrenal mass (mg)	28.8 ± 2.3	51.7 ± 18.9
Adrenal to body mass (mg/g)	0.06 ± 0.01	0.11 ± 0.04^§^

Data are means ± standard error (SE)

^*^Significantly different (*P* < 0.005) from cage control animals

^§^Significantly different (*P* < 0.05) from cage control animals

#### Effect of 28 days of hindlimb unloading on SREBP-2 and LDL-R in skeletal muscle

**Fig. 1:**
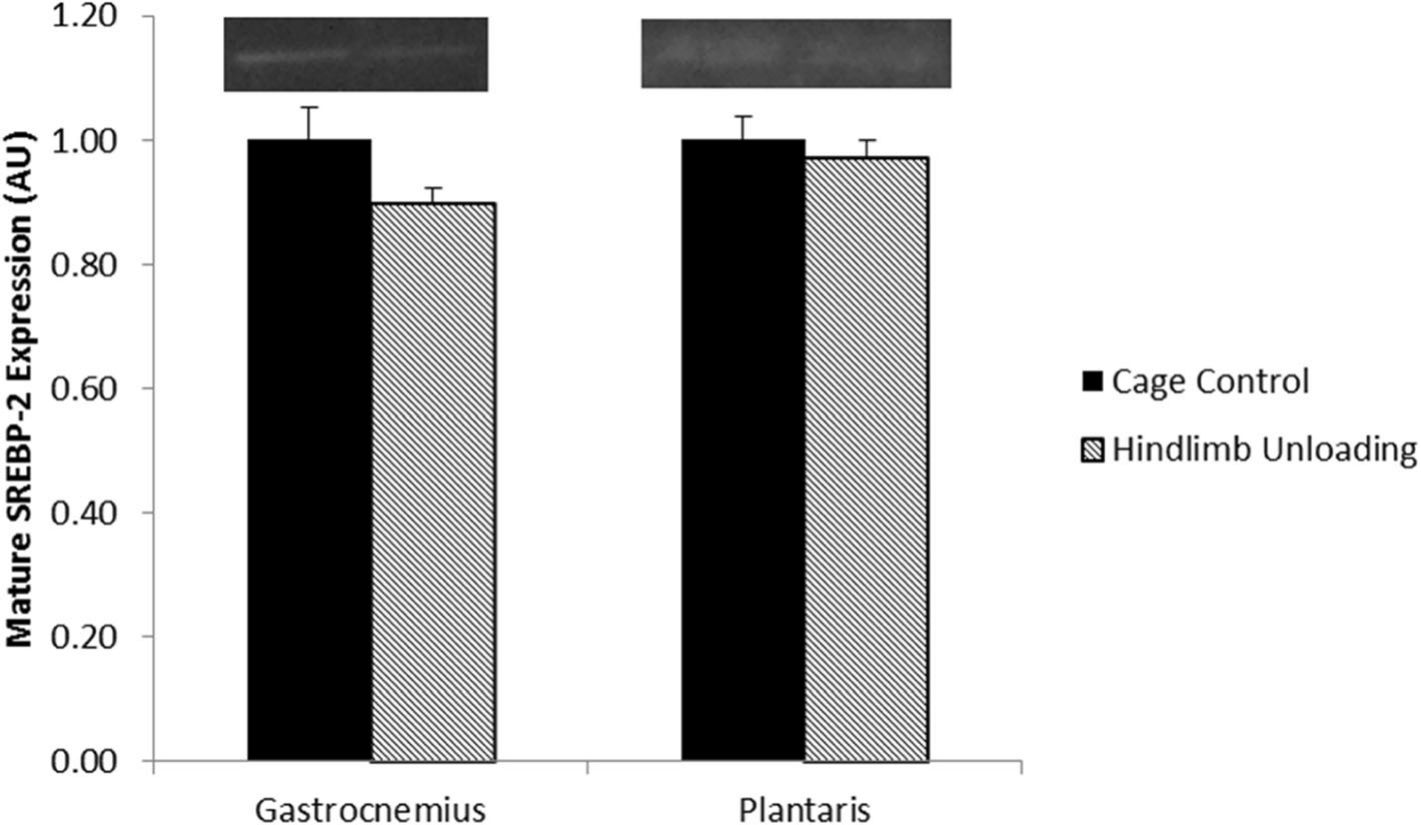
The effect of 28 days of hindlimb unloading on mature sterol regulatory element-binding protein-2 (SREBP-2) in gastrocnemius and plantaris muscles. Data are means ± standard error (SE). Cage Control (*n = 7*). Hindlimb Unloading (*n = 6*)

**Fig. 2:**
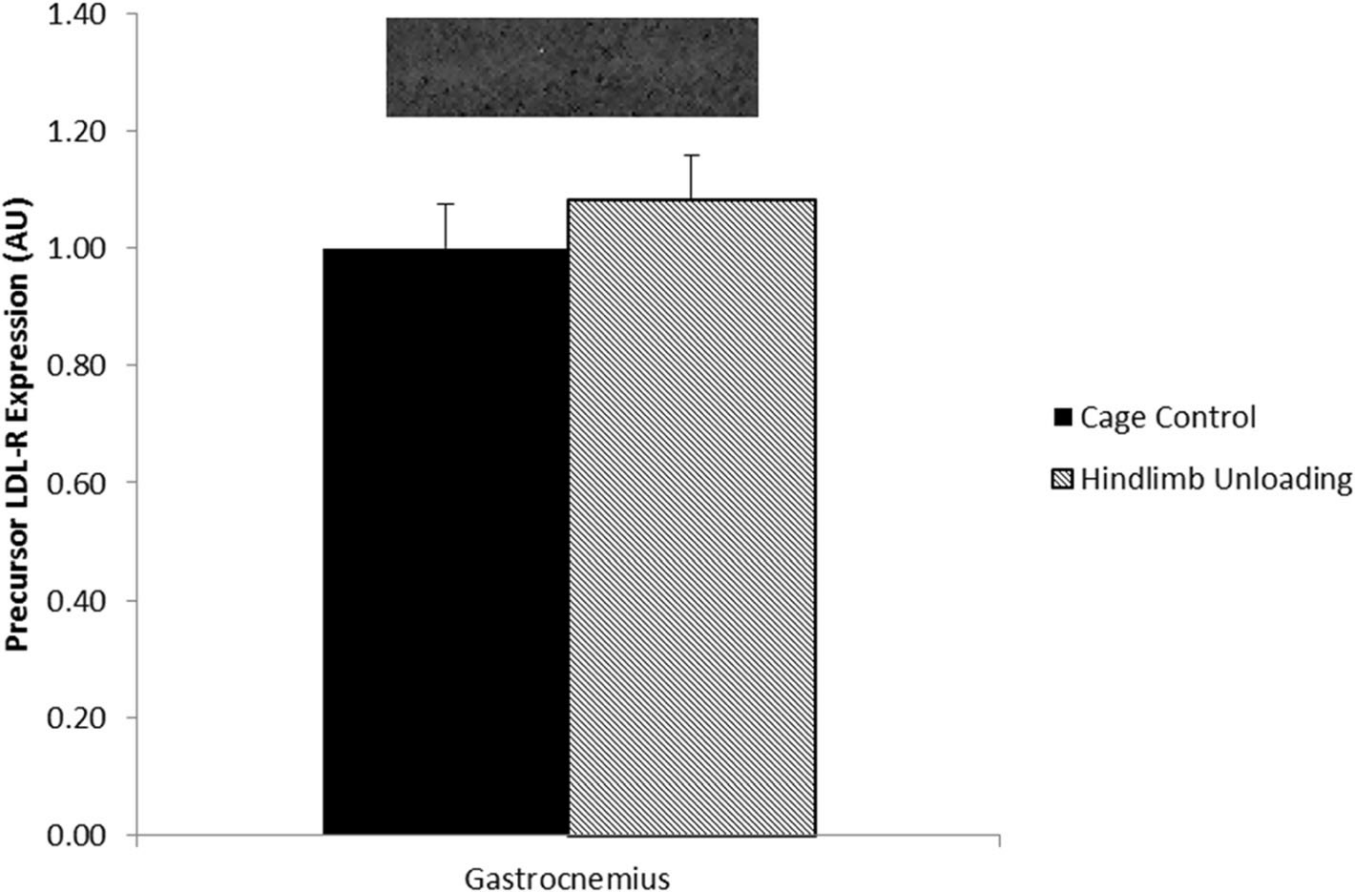
The effect of 28 days of hindlimb unloading on precursor low-density lipoprotein-receptor (LDL-R) in gastrocnemius muscle. Data are means ± standard error (SE). Cage Control (*n = 6)*. Hindlimb Unloading (*n = 4*)

There were no significant differences between groups in mature SREBP-2 for the gastrocnemius (*p* = 0.14) or plantaris muscles (*p* = 0.56) (Fig. 1). Similarly, there was no significant difference between groups in precursor LDL-R in the gastrocnemius muscle (*p* = 0.48) (Fig. 2).

### Protocol 2

#### Effect of dietary cholesterol and/or resistance training on body mass and composition, food intake and organ masses

At baseline, there was no significant difference in body mass among groups (Table 2). After 5 weeks, body mass was significantly less in the RT animals compared to the RTC groups, with no effect of dietary cholesterol. Furthermore, weight gain was significantly lower in the RT rats compared to the RTC and cage control animals. Food intake in the RT and RTC groups were significantly less than the cage control rats.

**Table 2:** Effect of dietary cholesterol and/or resistance training on body mass, food intake, and body composition

Variable		CC	RTC	RT
Baseline body mass (g)	No CL	408.0 ± 6.7	418.0 ± 10.3	403.9 ± 10.4
	CL	404.5 ± 10.0	412.5 ± 11.4	400.1 ± 12.0
Post-study body mass (g)	No CL	443.4 ± 14.4	463.1 ± 8.1	404.7 ± 16.7^*^
	CL	444.9 ± 12.5	444.2 ± 15.3	417.6 ± 15.1^*^
Average daily food intake (g/day)	No CL	24.1 ± 0.6	21.7 ± 0.9^§^	20.4 ± 0.8^§^
	CL	24.0 ± 0.3	22.8 ± 0.8^§^	21.4 ± 0.4^§^
Baseline lean mass (g)	No CL	338.6 ± 4.5	345.7 ± 9.8	338.8 ± 7.4
	CL	353.5 ± 15.7	344.8 ± 7.0	327.1 ± 9.4
Post-study lean mass (g)	No CL	350.9 ± 8.6	375.7 ± 10.7	336.3 ± 9.9^*^
	CL	356.5 ± 4.9	362.0 ± 11.5	343.1 ± 12.6^*^
Baseline fat mass (g)	No CL	43.6 ± 2.6	50.6 ± 6.2	46.0 ± 3.4
	CL	43.3 ± 4.4	45.4 ± 4.6	43.8 ± 2.4
Post-study fat mass (g)	No CL	72.3 ± 6.3	54.9 ± 5.9^§^	42.8 ± 6.1^§^
	CL	63.8 ± 4.5	51.8 ± 4.7^§^	43.9 ± 4.4^§^

Data are means ± standard error (group size n). CC = Cage Control. RTC = Resistance training control. RT = Resistance training. CL = Cholesterol. *n = 4–8* per group

^*^Significantly different (*P* < 0.05) from RTC groups

^§^Significantly different (P < 0.05) from CC groups

Based on DEXA data, there were no significant differences in total lean or fat mass among groups at baseline. However, at the end of the study, the RT animals had significantly less total lean mass than the RTC groups while the RTC and RT rats had significantly lower body fat compared to the cage control groups.

Soleus, soleus to body mass ratio, gastrocnemius, gastrocnemius to body mass ratio, and plantaris were not significantly different among groups (Table 3). However, plantaris to body mass ratio was significantly higher in the RT rats than the cage control animals. Adrenal mass and adrenal mass to body mass ratio were significantly higher in the RTC and RT groups compared to the cage control rats. It should be noted that gastrocnemius to body mass ratio and adrenal mass failed Levene’s test for homogeneity of variances.

**Table 3:** Effect of dietary cholesterol and/or resistance training on muscle and adrenal mass

Variable		CC	RTC	RT
Gastrocnemius (mg)	No CL	2250.5 ± 56.3	2305.4 ± 60.4	2058.6 ± 120.0
	CL	2318.9 ± 65.5	2300.9 ± 80.8	2191.7 ± 107.7
Gastrocnemius to BM (mg/g)	No CL	5.12 ± 0.12	4.98 ± 0.07	5.18 ± 0.19
	CL	5.21 ± 0.06	5.19 ± 0.10	5.32 ± 0.09
Soleus (mg)	No CL	183.9 ± 13.3	185.6 ± 7.4	168.9 ± 9.7
	CL	183.8 ± 10.2	183.6 ± 10.6	174.3 ± 8.7
Soleus to BM (mg/g)	No CL	0.42 ± 0.05	0.40 ± 0.01	0.42 ± 0.01
	CL	0.41 ± 0.02	0.41 ± 0.02	0.42 ± 0.01
Plantaris (mg)	No CL	452.3 ± 20.6	487.4 ± 13.8	444.9 ± 19.6
	CL	462.3 ± 9.1	472.1 ± 17.4	460.8 ± 20.1
Plantaris to BM (mg/g)	No CL	1.03 ± 0.05	1.05 ± 0.02	1.12 ± 0.03^*^
	CL	1.04 ± 0.02	1.07 ± 0.03	1.12 ± 0.02^*^
Adrenal mass (mg)	No CL	39.3 ± 7.0	63.9 ± 10.5^*^	50.9 ± 4.5^*^
	CL	38.3 ± 2.7	65.1 ± 7.1^*^	64.4 ± 2.3^*^
Adrenal to BM (mg/g)	No CL	0.09 ± 0.01	0.14 ± 0.02^*^	0.13 ± 0.01^*^
	CL	0.09 ± 0.01	0.14 ± 0.01^*^	0.16 ± 0.01^*^

Data are means ± standard error (group size n). *CC* Cage Control, *RTC* Resistance training control, *RT* Resistance training, *CL* Cholesterol, *BM* Body mass. *n = 4–8* per group

^*^Significantly different (P < 0.05) from CC groups

#### Effect of dietary cholesterol and/or resistance training on fractional synthesis rates

**Fig. 3:**
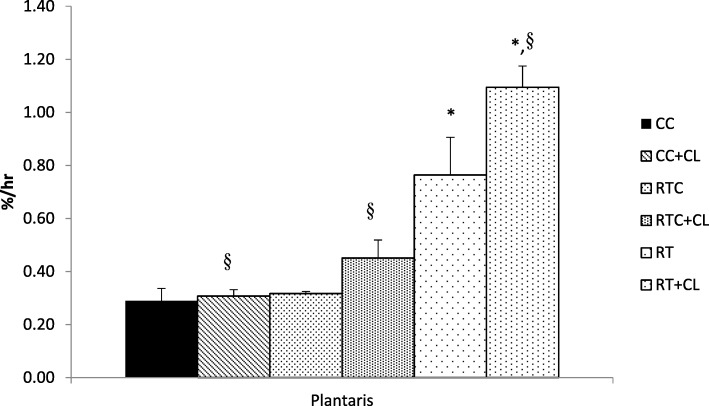
The effect of dietary cholesterol and/or resistance training on plantaris fractional synthesis rates. Data are means ± standard error (SE). *n = 3–5* per group. ^*^Significantly different (*P* < 0.05) from CC and RTC groups. §Significantly different (*P* < 0.05) from No CL groups

**Fig. 4:**
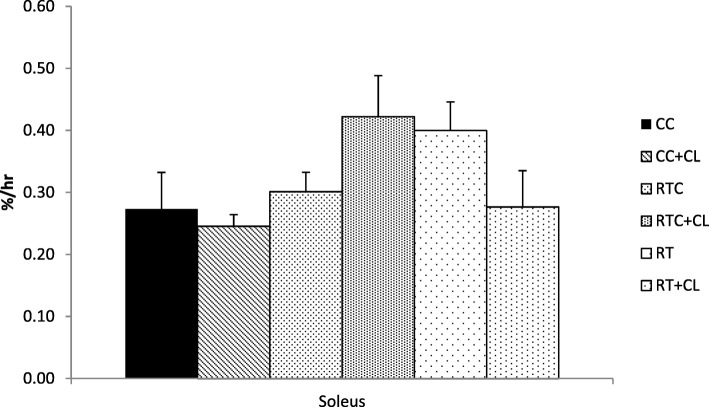
The effect of dietary cholesterol and/or resistance training on soleus fractional synthesis rates. Data are means ± standard error (SE). *n = 4–5* per group

After 5 weeks of exercise, the RT groups had significantly higher plantaris mixed muscle FSR than the RTC and CC animals (Fig. 3). Furthermore, there was a significant effect of cholesterol, with the CL groups showing greater plantaris mixed muscle FSR than the No CL rats. There was no significant difference among groups for soleus mixed muscle FSR (Fig. 4). It should be noted that plantaris FSR failed both Levene’s test for homogeneity of variances and the Shapiro-Wilk test of normality.

#### Effect of dietary cholesterol and/or resistance training on SREBP-2 and LDL-R in skeletal muscle

**Fig. 5:**
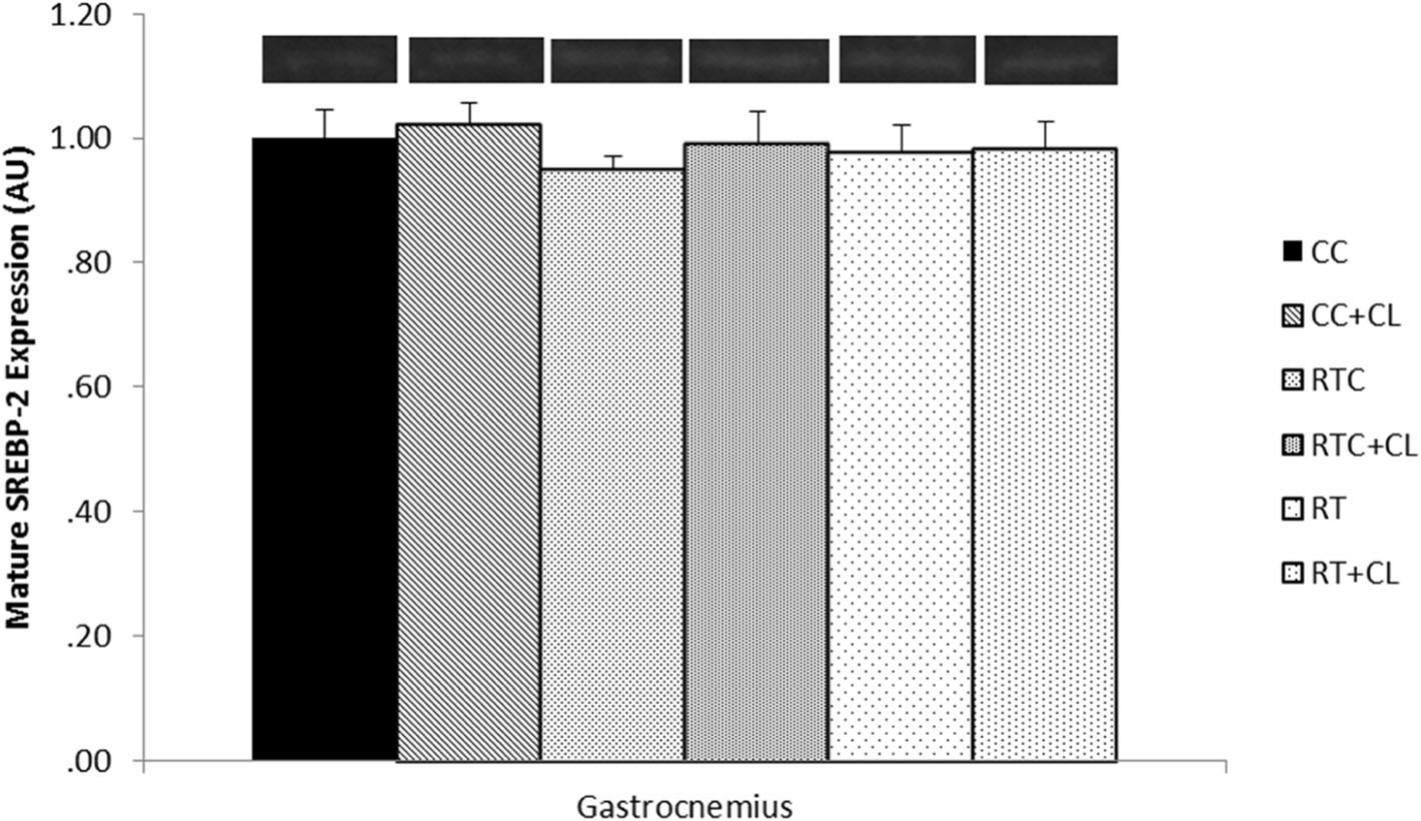
The effect of dietary cholesterol and/or resistance training on mature sterol regulatory element-binding protein-2 (SREBP-2) in gastrocnemius muscle. Data are means ± standard error (SE). *n = 3–8* per group

**Fig. 6:**
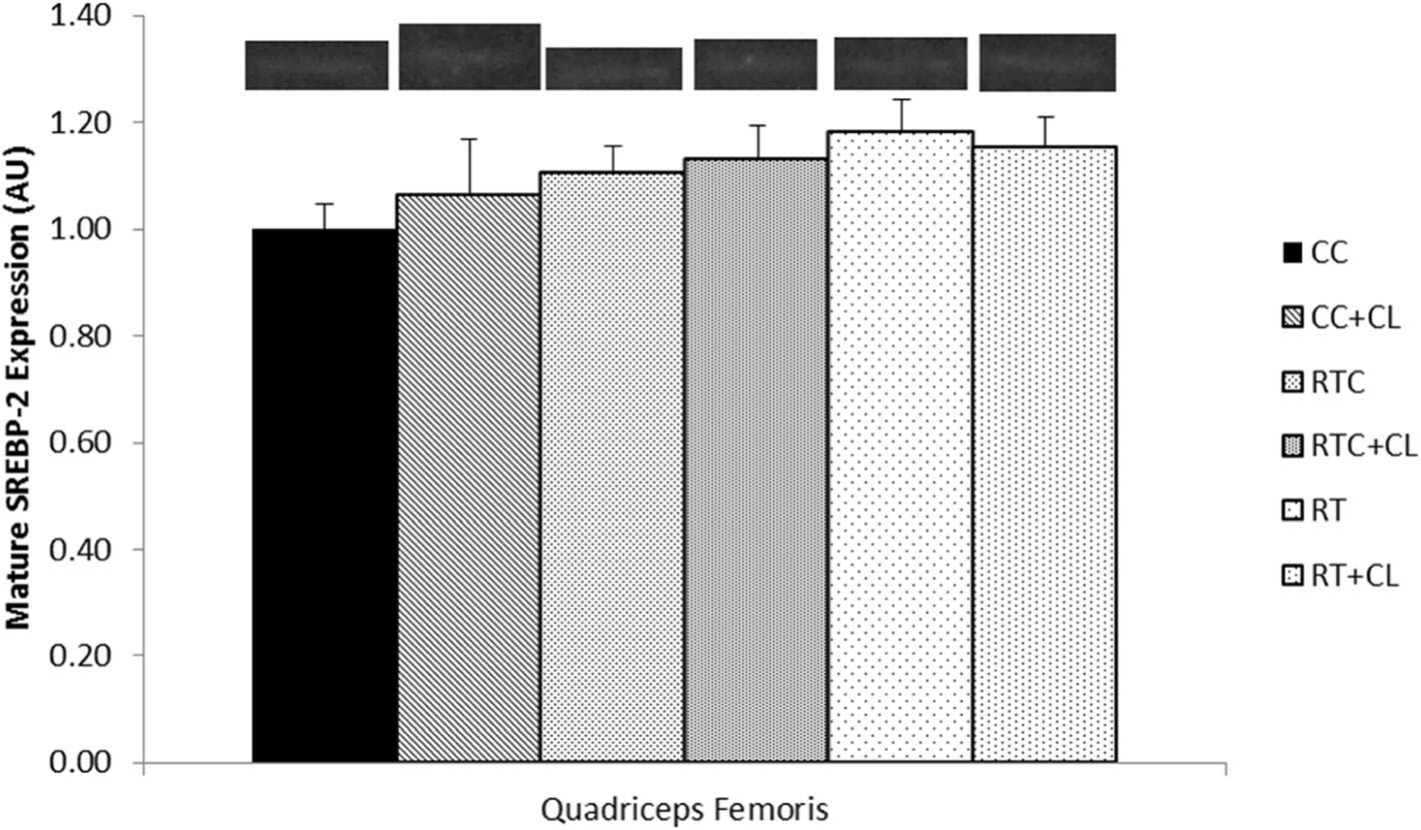
The effect of dietary cholesterol and/or resistance training on mature sterol regulatory element-binding protein-2 (SREBP-2) in quadriceps femoris muscle. Data are means ± standard error (SE). *n = 4–8* per group

**Fig. 7:**
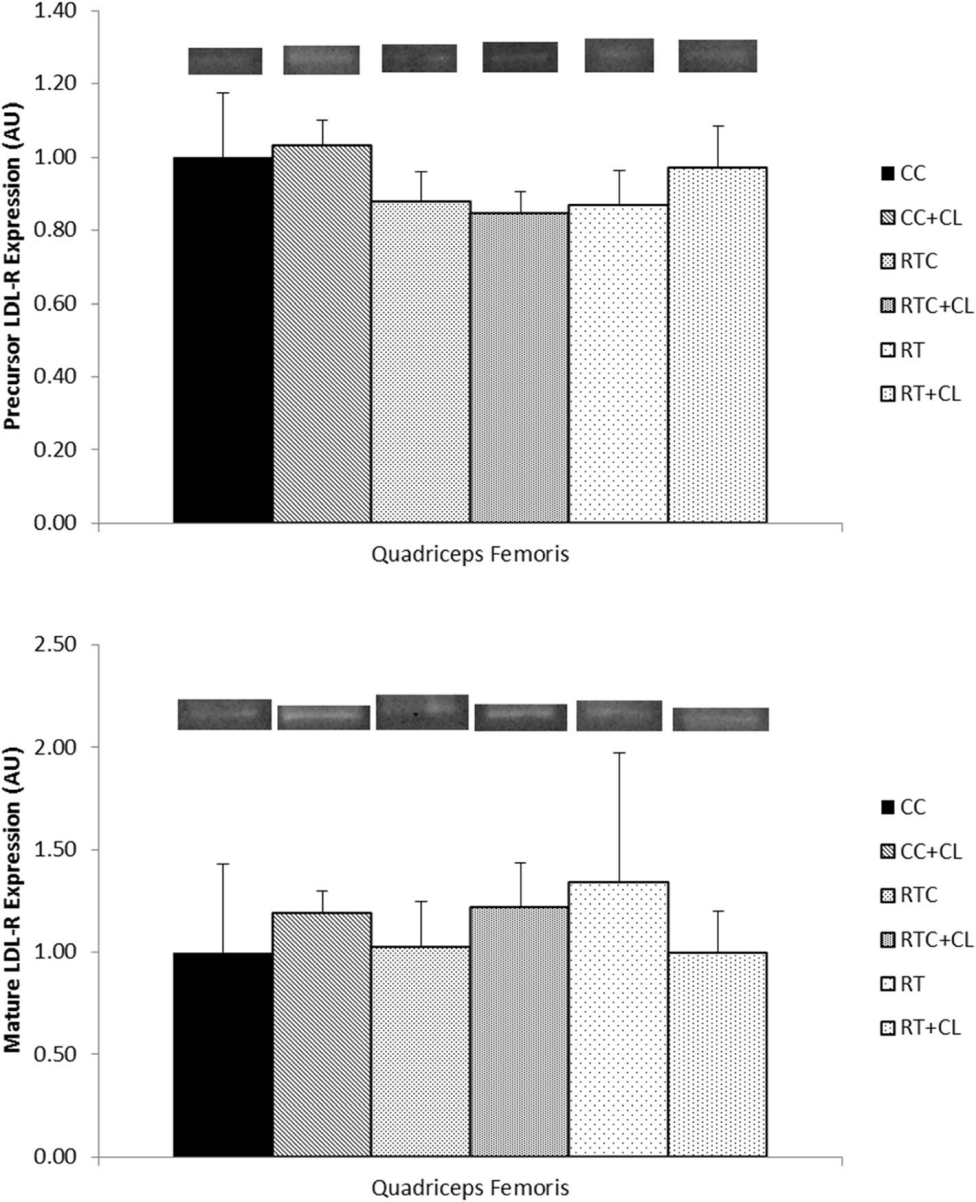
The effect of dietary cholesterol and/or resistance training on low-density lipoprotein-receptor (LDL-R) in quadriceps femoris muscle. **(a)** Precursor LDL-R. *n* = 3–6 per group (**b**) Mature LDL-R. Data are means ± standard error (SE). *n = 3–7* per group

Following 5 weeks of exercise, there was no significant difference among groups for mature SREBP-2 in the red gastrocnemius (Fig. 5) or quadriceps femoris (Fig. 6) muscles. Similarly, there was no significant difference among groups for precursor (130 kDa) or mature LDL-R in the quadriceps femoris muscle (Fig. 7). It should be noted that mature LDL-R for the quadriceps femoris failed the Shapiro-Wilk test of normality.

## Discussion

The current study aimed to investigate the effects of opposing treatments on rat skeletal muscle to obtain a better understanding of skeletal muscle in different environments. In protocol 1, 28 days of unloading resulted in an expected decrease in body mass and a lower amount of hindlimb muscle mass than CC animals but no significant effect on SREBP-2 and LDL-R in rat skeletal muscle. While in protocol 2, 5 weeks of RT and dietary cholesterol did not promote the predicted gains in body and lean mass and elevation of proteins essential to cholesterol metabolism, but did effectively show higher protein synthesis in plantaris.

Following HU (protocol 1), change in body mass along with gastrocnemius and plantaris muscle masses and their muscle mass to body mass ratios were significantly lower in the HU animals. Several studies previously reported significant atrophy of the gastrocnemius and plantaris muscles following various durations of HU [14, 15, 18]. The gastrocnemius muscle mass to body mass ratios in the HU (4.20 mg/g) and CC (5.18 mg/g) animals in the current study were comparable to the ratios in a separate 28-day unloading study (HU: 4.05 mg/g; CC: 5.31 mg/g) [15]. Furthermore, the plantaris muscle mass to body mass ratios in the HU (0.93 mg/g) and CC (1.08 mg/g) rats also were similar (HU: 0.91 mg/g; CC: 1.08 mg/g) to that study’s ratios, demonstrating the effectiveness of the HU methodology utilized in this investigation.

After 5 weeks of training, the RT animals gained significantly less body and lean mass than the RTC groups. This attenuated increase may be attributed to two related issues. The exercise performed by the RT and RTC rats was stressful, as demonstrated by the significantly higher adrenal mass and adrenal mass to body mass ratio than the cage control animals. Second, the RT and RTC groups consumed on average, significantly less food per day than the cage control rats, with the RT animals consuming the least amount. In fact, the exercise may have contributed to the lower food intake, with the stress deterring the rats from consuming more food. Thus, the combination of exercise and lower food consumption may explain why the RT groups gained less lean and body mass than the RTC animals.

Despite the RT animals gaining the least amount of lean mass, the gastrocnemius, soleus and plantaris and their respective body mass ratios (except plantaris to body mass) did not show any significant differences among groups. Additionally, post-study total fat mass was significantly lower in the RT & RTC groups than the CC animals. Taken together, this exercise protocol did not enhance muscle size but did promote overall leanness for the RT & RTC groups. One study that utilized an exercise protocol similar to this study also reported no change in muscle mass for these particular muscles [24]. However, that study did not measure total lean mass. Future studies investigating RT in animals should include total body lean mass to provide an overall effect of the exercise protocol.

Dietary cholesterol did not appear to play a significant role in skeletal muscle gains with RT, but did with plantaris mixed muscle FSR. We observed significant gains in lean mass with greater dietary cholesterol intake following 12 weeks of RT in older adults [8]. Other investigators have reported no significant effect of dietary cholesterol on lean mass gains with RT [25]. Since the RT and RTC animals consumed less food than the cage control animals, the potential beneficial effects of dietary cholesterol following RT may not have been observed in this study. Future studies should address this issue of food intake.

Following 5 weeks of exercise, the RT groups had significantly higher plantaris mixed muscle FSR and plantaris to body mass ratio than the CC animals. Since fast twitch fibers are more responsive to RT, predominantly fast twitch muscles such as the plantaris muscle are more likely to hypertrophy in response to training than slow twitch muscles (soleus) [26, 27]. From one study on acute RE, both plantaris and soleus FSR were not significantly different between exercising rats and cage control animals [23]. Those results, combined with our data, suggest that repeated resistance exercise is necessary to observe changes in FSR.

In the first protocol, mature SREBP-2 protein levels in the gastrocnemius and plantaris muscles were not significantly different between groups. However, there appeared to be a trend (*p* = 0.14) towards significance for mature SREBP-2 in the gastrocnemius muscle, with HU rats expressing lower levels. In a previous study investigating the liver of rats undergoing 14 days of HU, mature SREBP-2 expression was reported to be elevated in comparison to CC animals [28]. Those results, in combination with our data, present contrasting effects of HU on cholesterol metabolism in the liver and muscle. In the liver, since HU rats have been reported to have higher hepatic cholesterol synthesis than control animals, the elevated mature SREBP-2 expression in unloaded rats may lead to higher cholesterol synthesis [28]. In the gastrocnemius muscle, the lower levels of mature SREBP-2 in the HU animals compared to CC animals may reflect a diminished need for cholesterol in the atrophied muscle. Since skeletal muscle is a relatively low endogenous producer of cholesterol, SREBP-2 activation should target uptake more than biosynthesis [29, 30]. Lower levels of mature SREBP-2 may lead to less cholesterol uptake, which may partially explain the elevated blood cholesterol levels observed in HU rats in a previous study [28]. In one study investigating the effects of resistance exercise on mRNA levels in skeletal muscle, SREBP-2 mRNA was reported to be elevated immediately postexercise in men [31]. In our study, SREBP-2 was not significantly different among groups in either the red gastrocnemius or quadriceps femoris muscles following 5 weeks of training. However, there appeared to be a trend (*p* = 0.17) towards higher mature SREBP-2 with greater activity in the quadriceps femoris. The greater amount of SREBP-2 may lead to higher levels of cholesterol in muscle to facilitate repair following exercise. These contrasting results demonstrate the importance of skeletal muscle activity on SREBP-2 and possibly cholesterol. Future studies should further explore the effects of RE and unloading on SREBP-2 and cholesterol.

After 28 days of hindlimb suspension, there was no difference in the protein expression of precursor (130 kDa) LDL-R in the gastrocnemius muscle between HU and CC groups. During western blot analysis, the LDL-R may separate into a mature (160 kDa) form and two precursor forms (110 kDa and 130 kDa). In this study, the 110 kDa precursor and 160 kDa mature LDL-R could not be detected with our technique. However, disuse has been compared to aging since both conditions are associated with decrements in muscle mass [2]. In one aging study, the gastrocnemius muscle of older rats exhibited significantly lower mature LDL-R when compared with younger rats [29]. Since muscle mass is lowered in both conditions, it is possible that the reduction in mature LDL-R protein content may occur in unloading as well, coinciding with a diminished need for cellular cholesterol and consequently a decrease in SREBP-2 protein levels. The reduced uptake of cholesterol by LDL-R could be problematic as this may lead to elevated LDL cholesterol and consequently, increased risk for cardiovascular disease [32].

In the present study, LDL-R was not affected by dietary cholesterol or RT. Following 5 weeks of training, there were no significant differences among groups in either precursor (130 kDa) or mature LDL-R protein content in the quadriceps femoris. In one study, animals consuming normal or high cholesterol diets did not show significant difference in hepatic LDL-R activity or protein content [33]. These data suggest that dietary cholesterol or RT might not play a significant role in promoting LDL-R protein content and that the amount of LDL-R available is sufficient to meet the needs of each environment.

One limitation of these studies was the inability to measure total cholesterol content within the cell. Since only SREBP-2 and LDL-R protein expressions were measured, the effects of these proteins on cholesterol homeostasis were not directly observed and can only be hypothesized. Future studies assessing cholesterol content within the cell in conjunction with measuring the expression of these essential proteins should be considered.

## Conclusions

In summary, 28 days of HU resulted in significantly lower muscle mass in HU rats compared with CC animals. Furthermore, HU animals exhibited a trend towards lower mature levels of SREBP-2 in the predominantly fast glycolytic gastrocnemius muscle. However, unloading did not significantly affect protein levels of mature SREBP-2 in the predominantly fast oxidative plantaris or precursor LDL-R in the gastrocnemius muscles of HU animals when compared with CC rats. On the other spectrum, 5 weeks of exercise led to greater gains in lean and body mass in the RTC rats than RT animals but higher plantaris to body mass ratio and mixed muscle FSR in the RT than the RTC (plantaris FSR) and CC (both) animals. Moreover, the groups consuming cholesterol had greater plantaris FSR than the rats not consuming cholesterol. Mature SREBP-2 in quadriceps femoris exhibited a trend where the RT groups were greater than the CC animals. However, mature SREBP-2 in red gastrocnemius muscle and LDL-R protein levels in the quadriceps femoris were not significantly different among groups. Thus, these studies provide evidence for a relationship between skeletal muscle and cholesterol metabolism, but the exact nature of that association remains unclear.

## Abbreviations

ANOVA: analysis of variance
CC + CL: cage control consuming cholesterol group
CC: cage control group
CL: dietary cholesterol
DEXA: dual energy x-ray absorptiometry
FSR: fractional synthesis rate
HU: hindlimb unloading; hindlimb unloading group
IDV: integrated density value
LDL: low-density lipoprotein
LDL-R: low-density lipoprotein-receptor
RE: resistance exercise
RT + CL: resistance training consuming cholesterol group
RT: resistance training; resistance training group
RTC + CL: resistance training control consuming cholesterol group
RTC: resistance training control group
SAS: Statistical Analysis System
SE: standard error
SREBP-2: sterol regulatory element-binding protein-2

## References

[1] Flakoll P, Sharp R, Baier S, Levenhagen D, Carr C, Nissen S (2004). Effect of β-hydroxy-β-methylbutyrate, arginine, and lysine supplementation on strength, functionality, body composition, and protein metabolism in elderly women. Nutrition.

[2] Hunter GR, McCarthy JP, Bamman MM (2004). Effects of resistance training on older adults. Sports Med.

[3] LeBlanc A, Lin C, Shackelford L, Sinitsyn V, Evans H, Belichenko O, Schenkman B, Kozlovskaya I, Oganov V, Bakulin A, Hedrick T, Feeback D (2000). Muscle volume, MRI relaxation times (T2), and body composition after spaceflight. J Appl Physiol.

[4] Phillips SM, Tipton KD, Aarsland A, Wolf SE, Wolfe RR (1997). Mixed muscle protein synthesis and breakdown after resistance exercise in humans. Am J Phys.

[5] Rasmussen BB, Tipton KD, Miller SL, Wolf SE, Wolfe RR (2000). An oral essential amino acid-carbohydrate supplement enhances muscle protein anabolism after resistance exercise. J Appl Physiol.

[6] Holm L, Olesen JL, Matsumoto K, Doi T, Mizuno M, Alsted TJ, Mackey AL, Schwarz P, Kjær M (2008). Protein-containing nutrient supplementation following strength training enhances the effect on muscle mass, strength, and bone formation in postmenopausal women. J Appl Physiol.

[7] Fiatarone MA, O'Neill EF, Ryan ND, Clements KM, Solares GR, Nelson ME, Roberts SB, Kehayias JJ, Lipsitz LA, Evans WJ (1994). Exercise training and nutritional supplementation for physical frailty in very elderly people. N Engl J Med.

[8] Riechman SE, Andrews RD, Maclean DA, Sheather S (2007). Statins and dietary and serum cholesterol are associated with increased lean mass following resistance training. J Gerontol A Biol Sci Med Sci.

[9] Simons K, Ikonen E (2000). How cells handle cholesterol. Science.

[10] Riechman SE, Lee CW, Chikani G, Chen VC, Lee TV (2009). Cholesterol and skeletal muscle health. World Rev Nutr Diet.

[11] Espenshade PJ, Hughes AL (2007). Regulation of sterol synthesis in eukaryotes. Annu Rev Genet.

[12] Fluckey JD, Dupont-Versteegden EE, Knox M, Gaddy D, Tesch PA, Peterson CA (2004). Insulin facilitation of muscle protein synthesis following resistance exercise in hindlimb-suspended rats is independent of a rapamycin-sensitive pathway. Am J Physiol Endocrinol Metab.

[13] Fluckey JD, Dupont-Versteegden EE, Montague DC, Knox M, Tesch P, Peterson CA, Gaddy-Kurten D (2002). A rat resistance exercise regimen attenuates losses of musculoskeletal mass during hindlimb suspension. Acta Physiol Scand.

[14] Hurst JE, Fitts RH (2003). Hindlimb unloading-induced muscle atrophy and loss of function: protective effect of isometric exercise. J Appl Physiol.

[15] Knox M, Fluckey JD, Bennett P, Peterson CA, Dupont-Versteegden EE (2004). Hindlimb unloading in adult rats using an alternative tail harness design. Aviat Space Environ Med.

[16] Morey-Holton ER, Globus RK (2002). Hindlimb unloading rodent model: technical aspects. J Appl Physiol.

[17] Riley DA, Slocum GR, Bain JL, Sedlak FR, Sowa TE, Mellender JW (1990). Rat hindlimb unloading: soleus histochemistry, ultrastructure, and electromyography. J Appl Physiol.

[18] Zhang LF, Sun B, Cao XS, Liu C, Yu ZB, Zhang LN, Cheng JH, Wu YH, Wu XY (2003). Effectiveness of intermittent -Gx gravitation in preventing deconditioning due to simulated microgravity. J Appl Physiol.

[19] Swift JM, Swift SN, Allen MR, Bloomfield SA (2014). Beta-1 adrenergic agonist treatment mitigates negative changes in cancellous bone microarchitecture and inhibits osteocyte apoptosis during disuse. PLoS One.

[20] Smith PK, Krohn RI, Hermanson GT, Mallia AK, Gartner FH, Provenzano MD, Fujimoto EK, Goeke NM, Olson BJ, Klenk DC (1985). Measurement of protein using bicinchoninic acid. Anal Biochem.

[21] Romero-Calvo I, Ocón B, Martínez-Moya P, Suárez MD, Zarzuelo A, Martínez-Augustin O, de Medina FS (2010). Reversible Ponceau staining as a loading control alternative to actin in Western blots. Anal Biochem.

[22] Dufner DA, Bederman IR, Brunengraber DZ, Rachdaoui N, Ismail-Beigi F, Siegfried BA, Kimball SR, Previs SF (2005). Using 2H2O to study the influence of feeding on protein synthesis: effect of isotope equilibration in vivo vs. in cell culture. Am J Physiol Endocrinol Metab.

[23] Gasier HG, Riechman SE, Wiggs MP, Previs SF, Fluckey JD (2009). A comparison of 2H2O and phenylalanine flooding dose to investigate muscle protein synthesis with acute exercise in rats. Am J Physiol Endocrinol Metab.

[24] Farrell PA, Fedele MJ, Hernandez J, Fluckey JD, Miller JL, Lang CH, Vary TC, Kimball SR, Jefferson LS (1999). Hypertrophy of skeletal muscle in diabetic rats in response to chronic resistance exercise. J Appl Physiol.

[25] Iglay HB, Apolzan JW, Gerrard DE, Eash JK, Anderson JC, Campbell WW (2009). Moderately increased protein intake predominately from egg sources does not influence whole body, regional, or muscle composition responses to resistance training in older people. J Nutr Health Aging.

[26] Anderson JL, Aagaard P (2010). Effects of strength training on muscle fiber types and size; consequences for athletes training for high-intensity sport. Scand J Med Sci Sports.

[27] Armstrong RB, Phelps RO (1984). Muscle fiber type composition of the rat hindlimb. Am J Anat.

[28] Vecchini A, Ceccarelli V, Orvietani P, Caligiana P, Susta F, Binaglia L, Nocentini G, Riccardi C, Di Nardo P (2003). Enhanced expression of hepatic lipogenic enzymes in an animal model of sedentariness. J Lipid Res.

[29] Segatto M, Trapani L, Marino M, Pallottini V (2011). Age- and sex-related differences in extra-hepatic low-density lipoprotein receptor. J Cell Physiol.

[30] Spady DK, Dietschy JM (1983). Sterol synthesis in vivo in 18 tissues of the squirrel monkey, Guinea pig, rabbit, hamster, and rat. J Lipid Res.

[31] Mahoney DJ, Safdar A, Parise G, Melov S, Fu M, MacNeil L, Kaczor J, Payne ET, Tarnopolsky MA (2008). Gene expression profiling in human skeletal muscle during recovery from eccentric exercise. Am J Physiol Regul Integr Comp Physiol.

[32] Griffin JD, Lichtenstein AH (2013). Dietary Cholesterol and Plasma lipoprotein profiles: randomized-controlled trials. Curr Nutr Rep.

[33] Roach PD, Balasubramaniam S, Hirata F, Abbey M, Szanto A, Simons LA, Nestel PJ (1993). The low-density lipoprotein receptor and cholesterol synthesis are affected differently by dietary cholesterol in the rat. Biochim Biophys Acta.

